# Real-world data on the management of pazopanib-induced liver toxicity in routine care of renal cell cancer and soft tissue sarcoma patients

**DOI:** 10.1007/s00280-023-04615-7

**Published:** 2023-12-17

**Authors:** K. Westerdijk, S. D. Krens, N. Steeghs, W. T. A. van der Graaf, E. T. T. L. Tjwa, H. Westdorp, I. M. E. Desar, N. P. van Erp

**Affiliations:** 1 Department of Medical Oncology, Research Institute for Medical Innovation, Radboud University Medical Center, P.O. Box 9101, Nijmegen, The Netherlands; 2 Department of Clinical Pharmacy, Research Institute for Medical Innovation, Radboud University Medical Center, Nijmegen, The Netherlands; 3 Department of Pharmacy and Clinical Pharmacology, Amsterdam UMC, University of Amsterdam, Amsterdam, The Netherlands; 4 Department of Medical Oncology, Netherlands Cancer Institute, Antoni van Leeuwenhoek Hospital, Amsterdam, The Netherlands; 5 Department of Medical Oncology, Erasmus MC Cancer Institute, Erasmus Medical Center Rotterdam, Rotterdam, The Netherlands; 6 Department of Gastroenterology and Hepatology, Research Institute for Medical Innovation, Radboud University Medical Center, Nijmegen, The Netherlands

**Keywords:** Pazopanib, Liver toxicity, Drug-induced liver injury, Therapeutic drug monitoring, Prednisolone

## Abstract

**Purpose:**

Pazopanib is known to cause liver toxicity. A relationship between pazopanib exposure and alanine transaminase elevations has been described in clinical trials. This study investigated the relation between pazopanib exposure and liver toxicity in real-world patients and evaluated the management of pazopanib-induced liver toxicity in routine care.

**Methods:**

A retrospective observational cohort study was performed in patients treated with pazopanib in whom pazopanib exposure was measured. The percentage of patients with and without liver toxicity during treatment with pazopanib was calculated as well as the average pazopanib exposure in both groups. Furthermore, the management of patients with liver toxicity was evaluated.

**Results:**

Liver toxicity was observed in 25 out of the 133 patients included (19%). Pazopanib exposure was comparable in patients with or without liver toxicity (27.7 mg/L versus 28.1 mg/L). Seven patients permanently discontinued pazopanib after the occurrence of liver toxicity. Of the remaining 18 patients, continuation or restart of pazopanib after liver toxicity was successful in 16 patients and half of these patients were able to safely continue pazopanib at the same dose as prior to liver toxicity for the remaining duration of treatment.

**Conclusion:**

Our study did not demonstrate a clear relationship between pazopanib exposure and the occurrence of pazopanib-induced liver toxicity. Half of the patients were able to safely continue or restart pazopanib treatment after liver toxicity and received the same dose as prior to drug withdrawal. Successful interventions to address pazopanib-induced toxicity in the clinic led to an algorithm for the management of pazopanib-induced liver toxicity.

**Supplementary Information:**

The online version contains supplementary material available at 10.1007/s00280-023-04615-7.

## Introduction

Pazopanib is an oral tyrosine kinase inhibitor (TKI) registered for the treatment of metastatic renal cell carcinoma (mRCC) and soft tissue sarcoma (STS) [1, 2]. It targets multiple kinase receptors, including vascular endothelial growth factor receptor 1, 2 and 3 (VEGFR1-3), platelet-derived growth factor receptor α and β (PDGFRα-β) and cytokine receptor (KIT) [3].

Many drugs can cause drug-induced liver injury (DILI) [4, 5]. DILI is classified as either direct or indirect [6]. Direct DILI is the result of a direct effect of the drug or its metabolites on the hepatocytes. An example of a drug that causes direct DILI is acetaminophen where the reactive metabolite, N-acetyl-p-benzoquinone imine (NAPQI) causes liver injury [7]. Indirect DILI is less predictable and occurs in a fraction of patients using the drug [8, 9]. DILI can vary from asymptomatic elevation in liver enzymes to acute liver failure. Pazopanib has been reported to induce liver toxicity. The FDA label of pazopanib includes a black box warning for hepatotoxicity and recommends to monitor liver function before start, at week 3 and every other week thereafter during the first two months of treatment [10]. Elevations in serum alanine transaminase (ALT) and aspartate transaminase (AST) were observed in more than half of the patients in clinical trials, sometimes leading to severe and even fatal liver function disorders [1, 2, 10, 11].

The mechanism behind pazopanib-induced liver toxicity has not yet been elucidated [12]. It has been hypothesized that pazopanib reactive aldehyde metabolites could be responsible, or that liver toxicity is caused by inhibition of ATB binding cassette subfamily B member 11 (bile salt export pump) [13–15]. Furthermore, polymorphisms in the hemochromatosis gene (*HFE*) might be associated with ALT elevations [16]. Others suggest that polymorphisms in the gene uridine diphosphate glucuronosyltransferase 1A1 (*UGT1A1*), resulting in disorders with conjugation of bilirubin, adds to the risk of developing pazopanib-induced liver toxicity [17]. Pazopanib is a *UGT1A1* inhibitor and especially patients with Gilbert syndrome, caused by a polymorphism in *UGT1A1*, have an increased risk of hyperbilirubinemia with the use of pazopanib. Finally, it is hypothesized that pazopanib-induced liver toxicity might be caused by autoimmune inflammation which can be treated and prevented with corticosteroids [18–20].

The recommended starting dose of pazopanib is 800 mg OD taken fasted, while 600 mg OD taken with food leads to equivalent exposure [21]. Adequate exposure is essential since a clear exposure–response relationship has been shown [22–24]. As a result, Therapeutic Drug Monitoring (TDM) to individualize the dose is increasingly used to improve the benefit-risk balance in patients treated with pazopanib. Especially for patients with STS, for whom treatment options are limited, it is important to continue pazopanib treatment for as long as possible. Suttle et al. demonstrated that patients with mRCC and a pazopanib trough concentration (C_trough_) > 20.5 mg/L had a median progression free survival (PFS) of 52.0 weeks, compared to a median PFS of 19.6 weeks in patients with a pazopanib C_trough_ < 20.5 mg/L [22]. This observation was confirmed by Verheijen et al. [23]. While no clear cut-off level of pazopanib exposure for the development of toxicity has been described in literature, multiple studies do show a relationship between increased pazopanib exposure and adverse events, including liver enzyme changes [3, 22, 25]. The current label of pazopanib recommends to interrupt pazopanib treatment when liver enzymes increase > 8 × upper limit of normal (ULN) and to restart pazopanib at a reduced dose of 400 mg OD (50%) upon normalization. Furthermore, if liver enzyme abnormalities persist or recur, permanent discontinuation of pazopanib treatment is recommended.

The occurrence of liver toxicity in patients treated with pazopanib and its relationship with pazopanib exposure has mainly been studied in registration studies, while clinical trial patients differ substantially from real-world patients [3, 22, 26]. Strict adherence to the current recommendations for the management of pazopanib-induced liver toxicity may interfere with optimal pazopanib exposure or even limit patient’s treatment options. The primary aim of this study was to investigate the association between pazopanib exposure and the occurrence of liver toxicity in patients with solid tumors treated in routine care. The secondary aims were to evaluate the management of pazopanib-induced liver toxicity in routine care and to provide guidance for physicians.

## Materials and methods

### Patients

This observational study was performed using clinical data of patients with solid tumors who were treated with pazopanib and of whom at least one pazopanib trough level was available between March 2013 and February 2020. The current study was approved by the institutional review board at the Radboudumc and a waiver was granted for use of routine care data (dossier number 2018-4617).

Clinical data were collected from the electronic health records for all patients and included data on baseline characteristics (such as age, gender, body mass index (BMI) and performance status), diagnosis, disease stage, presence of liver metastases, laboratory investigations prior to the start of pazopanib, treatment with pazopanib (starting dose, dose adjustments during treatment and whether pazopanib was administered with or without food) and potentially hepatotoxic co-medication (statins or acetaminophen). Furthermore, to assess the occurrence of liver toxicity and the association with pazopanib exposure, ALT, AST, bilirubin, serum albumin levels and pazopanib trough concentrations were collected from patient records after start of pazopanib treatment. Finally, data were collected on pazopanib treatment duration, whether treatment with pazopanib was interrupted due to the occurrence of liver toxicity, whether patients received corticosteroids for liver toxicity and the reason for stopping pazopanib treatment.

### Assessment of liver toxicity

Liver toxicity was defined as ALT and/or AST > 3 × ULN (or > 3 × baseline level of normal (BLN) in case baseline was abnormal) on pazopanib treatment. This definition is based on the warning for liver toxicity in the FDA label of pazopanib and is equal to a grade 2 elevation according to the Common Terminology Criteria for Adverse Events (CTCAE) of the National Cancer Institute, version 5.0. Recovery was defined as a decline in ALT and/or AST < 3 × ULN (≤ grade 1 according to CTCAE). The percentage of patients with and without liver toxicity and the median time until the occurrence of liver toxicity was determined.

### Pharmacokinetics

Pazopanib levels were measured in routine care. In routine care pazopanib levels are measured after reaching steady-state pharmacokinetics, on average ~ 4 weeks after start of treatment and thereafter at the discretion of the treating physician. Pazopanib plasma samples were collected 6 to 32 h after intake of pazopanib. C_trough_ levels were calculated using the approach of Wang et al. [27].

Pazopanib plasma concentrations were measured using a validated liquid chromatography tandem mass spectrometry assay comparable to the method earlier described by van Erp et al. [28].

### Association between occurrence of liver toxicity and pazopanib exposure

In patients with liver toxicity the average pazopanib C_trough_ was calculated over 8 weeks prior to the occurrence of liver toxicity (details Supplemental Method 1). The median time until the occurrence of liver toxicity was taken as a reference interval for the patients without liver toxicity for whom the average pazopanib C_trough_ over 8 weeks until the median time was also calculated. The pazopanib C_trough_ were compared between patients with and without liver toxicity. Furthermore, the pazopanib C_trough_ at the time of occurrence of liver toxicity in patients with liver toxicity was compared to average pazopanib C_trough_ in patients without liver toxicity.

According to the method used in the FDA pharmacology review, the association between pazopanib C_trough_ and the occurrence of ALT > 5 × ULN was determined [3].

For patients with liver toxicity who continued pazopanib treatment after the occurrence of liver toxicity, the median duration of pazopanib treatment after the development of liver toxicity was retrieved from the medical records. Also, the average pazopanib C_trough_ after the occurrence of liver toxicity was calculated for the remaining duration of treatment.

### Association between occurrence of liver toxicity and survival

Patients were divided into two groups depending on the occurrence of liver toxicity (yes/no). Explorative analyses were performed between the occurrence of liver toxicity for PFS and overall survival (OS). PFS was defined as the time from start of pazopanib treatment until discontinuation or death due to progressive disease (PD). Patients who did not experience PD were censored at the date of pazopanib discontinuation due to other causes or the date of last follow-up. OS was defined as the time from start of pazopanib treatment until the date of death. In case patients were still alive at the time of database closure, they were censored at the date of last follow-up. The relationship between the occurrence of liver toxicity and PFS and OS were separately explored for RCC and STS.

### Proposal for management of pazopanib-induced liver toxicity

As this was an observational study, management of pazopanib-induced liver toxicity was at the discretion of the treating physician. Based on pazopanib exposure data and clinical experiences, an algorithm for the treatment of pazopanib-induced liver toxicity for patients in routine care was developed.

### Statistical analysis

Statistical analyses were performed using IBM SPSS statistics for Windows, version 27.0 (IBM Corp., Armonk, NY, USA). Patient characteristics, laboratory investigations at baseline, the occurrence and management of liver toxicity were described using descriptive statistics. Categorical variables were compared using the Chi-square test. Continuous variables that were not normally distributed were compared using the unpaired *T*-test on log-transformed data. Logistic regression was used to identify associations between the patient characteristics age, BMI, performance status, tumor type, pre-treatment, pazopanib exposure, statin and acetaminophen use and the occurrence of liver toxicity (ALT > 3 × ULN). Furthermore, the association between the occurrence of ALT > 5 × ULN and pazopanib C_trough_ was investigated using logistic regression, according to FDA analyses. Patient characteristics that were identified as predictors in univariate analysis were taken forward to multivariate analysis.

Pazopanib C_trough_ in patients with or without liver toxicity were compared using the unpaired *T*-test on log-transformed data.

PFS and OS were estimated with the Kaplan–Meier method and differences between patients with or without liver toxicity were examined with the log-rank test. Outcomes with a *P*-value less than 0.05 were considered statistically significant.

## Results

### Patients

Of the 133 patients included in this study, 25 (19%) met the definition of liver toxicity. Patient characteristics for both patients with or without liver toxicity are presented in Table 1. The median age was 62 (range 23–85) years and most patients were male (67%). A total of 98 patients were diagnosed with RCC, 33 patients with STS and 2 patients with gynaecological malignancies. Only albumin level was significantly different between both groups, though not clinically relevant. None of the patients who started pazopanib intake with food (*n* = 18) developed liver toxicity.

**Table 1:** Baseline characteristics

	No liver toxicity (*N* = 108)	Liver toxicity (*N* = 25)	Overall (*N* = 133)
Age in years	63 (23–85)	61 (31–80)	62 (23–85)
Gender			
Female	35 (32.4)	9 (36.0)	44 (33.1)
Male	73 (67.6)	16 (64.0)	89 (66.9)
BMI	26.1 (17.2–43.8)	27.4 (19.5–36.7)	26.4 (17.2–43.8)
Karnofsky Performance Score	80 (50–100)	80 (60–100)	80 (50–100)
Type of tumor			
RCC	82 (75.9)	16 (64.0)	98 (73.7)
STS	24 (22.2)	9 (36.0)	33 (24.8)
Other^a^	2 (1.9)	0 (0.0)	2 (1.5)
IMDC risk classification (RCC)			
Favorable	12 (14.6)	4 (25.0)	16 (16.3)
Intermediate	48 (58.5)	10 (62.5)	58 (59.2)
Poor	20 (24.4)	2 (12.5)	22 (22.4)
Unknown	2 (2.4)	0 (0.0)	2 (2.0)
Presence of liver metastases^b^			
Yes	21 (19.6)	6 (24.0)	27 (20.5)
No	86 (80.4)	19 (76.0)	105 (79.5)
Pretreatment with systemic therapy			
Yes	52 (48.1)	12 (48.0)	64 (48.1)
No	56 (51.9)	13 (52.0)	69 (51.9)
Starting dose pazopanib			
800 mg without food	71 (65.7)	20 (80.0)	91 (68.4)
600 mg with food	15 (13.9)	0 (0.0)	15 (11.3)
600 mg without food	6 (5.6)	2 (8.0)	8 (6.0)
400 mg with food	3 (2.8)	0 (0.0)	3 (2.3)
400 mg without food	13 (12.0)	2 (8.0)	15 (11.3)
200 mg without food	0 (0.0)	1 (4.0)	1 (0.8)
Laboratory investigations			
Hemoglobin (mmol/l)	7.5 (4.3–10.3)	8.1 (5.0–9.7)	7.5 (4.3–10.3)
White blood cells (*10^9^/l)	7.3 (3.3–16.2)	7.0 (4.0–67.8)	7.2 (3.3–67.8)
Thrombocytes (*10^9^/l)	270 (38–787)	242 (134–685)	263 (38–787)
Neutrophils (*10^9^/l)	5.0 (1.7–15.0)	4.7 (2.4–8.8)	4.8 (1.7–15.0)
Calcium (mmol/l)	2.4 (1.6–3.1)	2.4 (2.3–2.6)	2.4 (1.6–3.1)
Creatinine (umol/l)	89 (40–355)	98 (55–216)	90 (40–355)
ALT (IU/l)	22 (9–106)	23 (16–64)	23 (9–106)
AST (IU/l)	23 (9–82)	24 (16–62)	24 (9–82)
LDH (IU/l)	212 (118–687)	197 (120–878)	209 (118–878)
GGT (IU/l)	49 (11–650)	40 (14–364)	47 (11–650)
ALP (IU/l)	101 (41–587)	88 (47–271)	97 (41–587)
Bilirubin, total (umol/l)	6 (3–22)	6 (4–13)	6 (3–22)
Bilirubin, direct (umol/l)	3 (2–16)	2 (2–6)	2 (2–16)
Albumin (g/l)*	33 (19–43)	37 (19–42)	34 (19–43)
Use of statin^b^			
Yes	19 (17.8)	6 (24.0)	25 (18.9)
No	88 (82.2)	19 (76.0)	107 (81.1)
Use of acetaminophen^b^			
Yes	80 (74.8)	17 (68.0)	97 (73.5)
No	27 (25.2)	8 (32.0)	35 (26.5)

Continuous variables are presented as median (range) and categorical variables as *n* (%), unless otherwise specified

*ALP* Alkaline phosphatase, *ALT* alanine transaminase, *AST* aspartate transaminase, *GGT* gamma-glutamyl transferase, *LDH* lactate dehydrogenase

*Statistically significant

^a^One patient had ovarian cancer and one patient had endometrial cancer

^b^Data were missing for one patient

### Development of liver toxicity

The median time until the occurrence of liver toxicity was 37 (interquartile range (IQR) 28–58) days. Seven patients (28%) had ALT/AST 3–5 × ULN, 4 patients (16%) ALT/AST 5–8 × ULN and 14 patients (56%) ALT/AST > 8 × ULN. Two of the patients with ALT/AST > 8 × ULN also had bilirubin level > 2 × ULN.

The pazopanib dose at which liver toxicity occurred was 800 mg fasted in 18/25 patients (72.0%). The remaining patients received a dose of 800 mg with food (1/25, 4.0%), 600 mg fasted (3/25, 12.0%), 600 mg with food (1/25, 4.0%) or 400 mg fasted (2/25, 8.0%). In 18/25 patients (72%) the dose at which liver toxicity occurred was the same as the starting dose, 5 patients had a dose escalation based on a pazopanib C_trough_ < 20.5 mg/L prior to developing liver toxicity and 2 patients a dose reduction based on tolerability issues, being non-liver toxicity.

### Association between pazopanib exposure and occurrence of liver toxicity

For patients with liver toxicity, pazopanib C_trough_ prior to the occurrence of liver toxicity (median 37 days) was available from 21/25 patients (84%). For patients without liver toxicity, the pazopanib C_trough_ was available from 98/108 (91%) patients. The median number of available pazopanib C_trough_ levels was 1 (range 0–2).

**Fig. 1:**
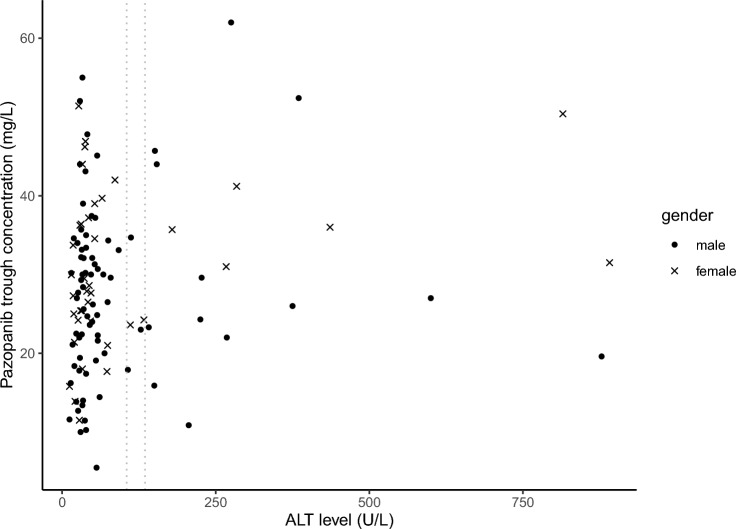
Average pazopanib concentration for individual patients in relation to ALT level. Scatter plot of the average pazopanib C_trough_ prior to the occurrence of liver toxicity in relation to ALT level for both males (dots) and females (crosses) (*n* = 119 patients). The vertical dotted grey lines represent the 3 × ULN for both males (right) and females (left). The average pazopanib C_trough_ was calculated over 8 weeks prior to the occurrence of liver toxicity for patients with liver toxicity and over 8 weeks until the median time to liver toxicity for patients without liver toxicity. *ALT* alanine transaminase

The average pazopanib C_trough_ (median (IQR)) prior to occurrence of liver toxicity was 27.7 (23.5–38.6) mg/L for patients with liver toxicity compared to 28.1 (21.1–34.6) mg/L for patients without liver toxicity (*P* = 0.335). Pazopanib C_trough_ at the moment of occurrence of liver toxicity was available from 22/25 patients (88%) (30.3 (23.5–41.9) mg/L) and did not differ from average pazopanib concentration in patients without liver toxicity (*P* = 0.146). The average pazopanib C_trough_ for each individual patient is shown in Fig. 1.

Logistic regression analysis demonstrated a significant association between the occurrence of ALT > 5 × ULN and average pazopanib C_trough_, with an odds ratio (OR) of 1.064 (95% confidence interval 1.010–1.122). Though the average pazopanib C_trough_ in patients with ALT > 5 × ULN was similar to patients with ALT < 5 × ULN (27.7 (21.3–34.6) mg/L versus 31.3 (25.0–43.5) mg/L; *P* = 0.136).

Logistic regression analysis demonstrated no statistically significant associations between the occurrence of liver toxicity and patients’ age, BMI, performance status, tumor type, pre-treatment, pazopanib exposure, statin or acetaminophen use.

### Management and follow-up of liver toxicity

**Fig. 2:**
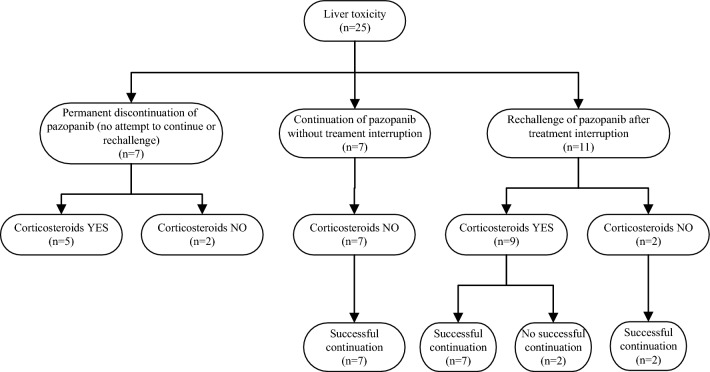
Management of liver toxicity in study cohort. Management of liver toxicity, including whether pazopanib was rechallenged or permanently discontinued when liver toxicity occurred. Reasons for permanent discontinuation without attempting to rechallenge were PD (*n* = 1), death due to a combination of PD and liver toxicity (*n* = 1), ALT/AST > 8 × ULN combined with a bilirubin level > 2 × ULN (*n* = 2) and switch to another line of treatment due to liver toxicity (*n* = 3). *PD* progressive disease; *TKI* tyrosine kinase inhibitor

The management and follow-up of patients with pazopanib-induced liver toxicity is shown in Fig. 2.

Of the 7 patients with ALT/AST 3–5 × ULN, 6 patients continued pazopanib without treatment interruption. The dose was increased in 1 patient (due to a low pazopanib C_trough_ of 15.9 mg/L), decreased in 1 patient (due to a high C_trough_ of 64.2 mg/L) and continued without dose alteration in 4 patients. In 1 patient pazopanib was interrupted and restarted after treatment with corticosteroids.

Of the 4 patients with ALT/AST 5–8 × ULN, 1 permanently discontinued pazopanib treatment due to PD. In the remaining 3 patients, pazopanib was interrupted and restarted after treatment with corticosteroids.

Of the 14 patients with ALT/AST > 8 × ULN, pazopanib was either continued without treatment interruption (*n* = 1, no dose alteration), restarted after treatment interruption (*n* = 7) or permanently discontinued (*n* = 6). Five of 7 patients who restarted pazopanib after treatment interruption were treated with corticosteroids for liver toxicity. In the 6 patients who permanently discontinued pazopanib, 5 patients received treatment with corticosteroids. Reasons for permanent discontinuation were ALT/AST > 8 × ULN combined with a bilirubin level > 2 × ULN (*n* = 2), death (*n* = 1; due to a combination of PD and liver toxicity) and switch to another line of treatment (*n* = 3; 2 patients switched to sunitinib and 1 to everolimus). Management of individual patients with pazopanib-induced liver toxicity is shown in Supplemental Table 1.

In total, 18 out of 25 patients (72%) continued/restarted pazopanib treatment after occurrence of liver toxicity. Pazopanib could be successfully continued in 16/18 patients (89%) for a prolonged period (median 231 days (66–1282) in RCC and 200 days (50–990) in STS)). The pazopanib dose after the occurrence of liver toxicity compared to the dose when liver toxicity occurred is shown in Table 2. Half of the patients were able to safely continue pazopanib after liver toxicity at the same dose as before the occurrence of liver toxicity. Average pazopanib C_trough_ after the occurrence of liver toxicity was 28.9 (21.0–47.4) mg/L. All patients who continued/restarted pazopanib treatment had adequate pazopanib C_trough_ (> 20.5 mg/L). Nine of 18 patients (50%) received treatment with corticosteroids, either due to lack of recovery of ALT (*n* = 4), recurrence of liver toxicity after restart of pazopanib (*n* = 2) or simultaneously with pazopanib restart (*n* = 3). Patients who started corticosteroids had higher ALT levels compared to patients without treatment with corticosteroids (median (IQR) 385 (247–452) IU/L versus 151 (122–361) IU/L; *P* = 0.08).

**Table 2:** Dosing patterns in patients experiencing liver toxicity

Change in dose at pazopanib continuation or restart	Number of patients (%)
Continued at the same dose	9 (50)
800 mg fasted	6 (33)
600 mg with food	2 (11)
600 mg fasted	1 (6)
Decrease in dose	8 (44)
1 dose level	7 (39)
600 mg with food	1 (6)
600 mg fasted	4 (22)
200 mg with food	1 (6)
200 mg fasted	1 (6)
2 dose levels	1 (6)
400 mg fasted	1 (6)
Increase in dose (1 dose level)	1 (6)
800 mg with food	1 (6)

Of all the patients with liver toxicity, 23 patients (92%) showed recovery of liver toxicity. One of the patients in whom there was no recovery died due to a combination of rapid disease progression and liver failure (probably due to extensive liver metastases) and 1 patient was lost to follow-up. Median time until recovery of liver toxicity was 21 (7–105) days.

Based on the observations made in this study and the recommendations listed in the drug label an expert opinion-based algorithm was developed for the management of pazopanib-induced liver toxicity (Table 3). The first step in the algorithm is the evaluation of ALT and bilirubin level. The second step is the evaluation of pazopanib C_trough_ at the occurrence of liver toxicity. Based on the clinical data several recommendation steps are incorporated thereafter.

**Table 3:** Algorithm to treat liver toxicity based on laboratory investigation and measured pazopanib exposure

Laboratory investigation	Pazopanib concentration	Recommendation–step 1	Recommendation–step 2	Recommendation–step 3	Recommendation–step 4	Recommendation for pazopanib dose at restart or continuation^b^
ALT > 3 × ULN + bili > 2 × ULN	–	Stop pazopanib				
ALT 3–5 × ULN	< 30 mg/L	Continue pazopanib + weekly monitoring liver enzymes	No recovery^a^ ⟶interrupt pazopanib until recovery	No recovery^a^/recurrence ⟶ corticosteroids	No recovery^a^/recurrence ⟶ stop pazopanib	No dose adjustment
ALT 3–5 × ULN	≥ 30 mg/L	Continue pazopanib + weekly monitoring liver enzymes	No recovery^a^ ⟶ interrupt pazopanib until recovery	No recovery^a^/recurrence ⟶ corticosteroids	No recovery^a^/recurrence ⟶ stop pazopanib	Consider − 1 dose level^c^
ALT > 5 × ULN	< 30 mg/L	Interrupt pazopanib until recovery	No recovery^a^/recurrence ⟶ corticosteroids	No recovery^a^/recurrence ⟶ stop pazopanib		No dose adjustment
ALT > 5 × ULN	≥ 30 mg/L	Interrupt pazopanib until recovery	No recovery^a^/recurrence ⟶ corticosteroids	No recovery^a^/recurrence ⟶ stop pazopanib		Consider −1 dose level^c^

Recommended corticosteroids schedule	
	Dose corticosteroids
Week 1	Prednisolone 30 mg OD
Week 2 + 3	Prednisolone 15 mg OD
Week 4 + 5 (if ALT < 3 × ULN)	Prednisolone 10 mg OD
Week 6 + 7 (if ALT persistently < 3 × ULN)	Prednisolone 5 mg OD
After week 7	Stop prednisolone

Dose levels of pazopanib		
Dose level	Pazopanib dose fasted^d^	Pazopanib dose with food^e^
– 3	200 mg OD fasted	–
− 2	400 mg OD fasted	200 mg OD with food
− 1	600 mg OD fasted	400 mg OD with food
0	800 mg OD fasted^f^	600 mg OD with food^f^
1	400 mg BID fasted	800 mg OD with food

*ALT* Alanine transaminase, *ULN* upper limit of normal, *ALT* alanine transaminase, *OD* once a day, *ULN* upper limit of normal, *BID* twice a day, *OD* once a day

^a^Recovery is defined as ALT < 3 × ULN

^b^Dose recommendations apply to every step

^c^− 1 dose level refers to a − 1 level dose reduction, regardless of the initial dose level

^d^Drug label pazopanib [10]

^e^Lubberman et al. [21]

^f^Bioequivalent dose

### Association between occurrence of liver toxicity and survival

The median (range) duration of follow-up for patients with or without liver toxicity was 20.6 (1.5–76.7) and 17.1 (1.0–85.6) months, respectively. For both RCC and STS, no statistically significant difference in both PFS and OS was observed between patients with or without liver toxicity (Supplemental Figs. 1 and 2).

## Discussion

In this retrospective observational cohort study, we investigated pazopanib-induced liver toxicity in real-world patients and developed a practical expert opinion-based algorithm for the management of pazopanib-induced liver toxicity, defined as ALT and/or AST > 3 × ULN (or > 3 × BLN). Pazopanib exposure was comparable in patients with or without liver toxicity. Continuation of pazopanib was attempted in 18/25 patients and successful in 16/18 patients. A total of 9/18 patients were able to safely continue pazopanib at the same dose as before the occurrence of liver toxicity. In total, 16 out of 25 patients were able to safely continue or resume pazopanib treatment despite liver toxicity.

This is the first study describing the association between pazopanib exposure and the occurrence of liver toxicity in real-world patients. In this real-world population, we investigated possible factors that are known to predispose individuals for DILI. Furthermore, we developed a practical algorithm to guide clinicians in the management of pazopanib-induced liver toxicity. In our study, approximately 20% of patients developed liver toxicity according to the definition in the drug label and similar to the percentages reported in the registration trials [1, 2]. We found no significant difference in average pazopanib C_trough_ between patients with or without liver toxicity. In contrast, Suttle et al. described that the incidence of ALT elevations increased with increasing pazopanib C_trough_ [22]. However, Noda et al. showed in a small study (*n* = 27), that pazopanib exposure was not significantly associated with grade ≥ 2 ALT elevation [29]. We did find an increased probability of ALT > 5 × ULN with increasing pazopanib steady state C_trough_, according to the FDA pharmacology review, with an OR of 1.064 which we consider not clinically relevant [3].

Pazopanib-induced liver toxicity is a treatment-limiting toxicity that can have important clinical implications, especially in patients with STS for whom treatment options are limited [30, 31]. The recommendations for the management of pazopanib-induced liver toxicity according to the drug label are limited to either interrupting or permanently discontinuing pazopanib treatment and, if possible, restarting pazopanib at a reduced dose [10]. This study clearly demonstrates the wide variety in the management of liver toxicity in real-world practice. Therefore, the recommendations in the drug label were combined with the observations in this study and translated into an expert opinion-based treatment algorithm for pazopanib-induced liver toxicity. For patients with a combination of ALT > 3 × ULN and bilirubin level > 2 × ULN pazopanib should be permanently discontinued [10]. For patients with ALT 3–8 × ULN the label recommends to continue pazopanib with weekly monitoring of ALT levels, and for patients with ALT > 8 × ULN to interrupt pazopanib. However, most medical oncologists are accustomed to use CTCAE grading to score and assess toxicity in clinical practice, in which a grade 2 ALT elevation is defined as ALT 3–5 × ULN and a grade 3 as ALT 5–20 × ULN [32]. Therefore, the cut-off of ALT > 5 × ULN was incorporated into the algorithm. In case treatment is interrupted, the drug label recommends to restart pazopanib at a reduced dose of 400 mg OD (50%). Since the limited relationship between pazopanib exposure and the occurrence of liver toxicity shown here, this advice is questionable, especially since pazopanib exposure has been related to PFS in mRCC [22, 33]. Therefore, patients with pazopanib-induced liver toxicity should maintain adequate pazopanib exposure for the remaining part of their treatment. To prevent unnecessary dose reductions leading to subtherapeutic exposure and thereby lack of efficacy, measurement of pazopanib C_trough_ at occurrence of liver toxicity was incorporated into the algorithm. A dose reduction should only be considered in patients with higher pazopanib C_trough_. A threshold for pazopanib exposure of > 30 mg/L was incorporated into the algorithm, based on the fact that a dose reduction at this concentration with 25–33% will still result in pazopanib exposure > 20.5 mg/L, which is in line with the observation in the current study that all patients who continued/restarted pazopanib treatment had adequate pazopanib C_trough_ (> 20.5 mg/L), regardless of the dose they received. Recovery of liver toxicity was defined as a decline in ALT < 3 × ULN. The definition of no recovery was at the discretion of the treating physician, however it concerned patients with a very slow decline, plateau or even rise in ALT level. Based on our experience in routine practice, we recommend to start treatment with prednisolone 30 mg OD in patients without recovery of ALT (3 × ULN) after interruption of pazopanib or patients with recurrence of ALT elevations after restart of pazopanib [18, 20]. The rationale behind corticosteroids is that pazopanib-induced liver toxicity might be immune mediated, as has been described for imatinib [34]. In immune-mediated DILI it is believed that drug metabolites activate the immune system and CD8 T lymphocytes [35]. It could have been interesting to investigate human leukocyte antigen (HLA) alleles, since HLA-B*57:01 carriage has been associated with liver toxicity [19]. However, due to the retrospective character of this study, it is not possible anymore to retrieve these data.

A remarkable finding in this study was that none of the 18 patients who started pazopanib intake with food developed liver toxicity. Patient numbers are small and there are no reports in literature describing this phenomenon for pazopanib or other hepatotoxic drugs. Further observations are needed to conclude if this food effect is relevant, and if it is, what the mechanism would be.

In the current study, although patient numbers were small, it appeared from the exploratory analyses that PFS and OS were not worse for patients with liver toxicity, neither predictive for efficacy, as is, for example,the onset of pazopanib-induced hand-foot syndrome in patients with STS [36].

Some factors are known to predispose individuals for DILI, such as obesity or pre-existing liver disease (including the presence of liver metastases) [37, 38]. A relationship between these factors and the occurrence of liver toxicity could not be confirmed in the current study, possibly due to small patient numbers. There might be other risk factors for the occurrence of liver toxicity, however, due to the limited number of patients in this study a selection of covariates was made to be included in the logistic regression analysis. Acetaminophen use was investigated as a possible covariate as well. However, acetaminophen is available as over-the-counter medication and may be used by patients without reporting it to their physician. As a limitation of a retrospective study, this was not documented explicitly in the patient files and could possibly have biased the results. However, since acetaminophen-induced DILI is most often the result of supratherapeutic dosages, it is unlikely that this has affected our results [7]. Other laboratory abnormalities apart from ALT, AST and bilirubin have been associated with liver toxicity as well, such as gamma-glutamyl transferase [39]. However, based on the drug label, ALT, AST and bilirubin were investigated in this study. Since this was an observational cohort study including real-world patients, the interruption of pazopanib or initiation of corticosteroids was performed on the initiative of the treating physician. Therefore, it is uncertain whether recovery of liver toxicity was the result of treatment with corticosteroids or part of the natural course of the disease. Finally, we only included patients of whom at least one pazopanib trough level was available. Especially during the first part of this study period, measurement of pazopanib C_trough_ was not yet incorporated into routine care and was especially used in patients experiencing adverse events or lacking efficacy of treatment, which could have biased the results.

## Conclusion

In this study, we did not find a relation between pazopanib exposure and the occurrence of liver toxicity. Our clinical experience based algorithm, based on dose alterations and treatment with corticosteroids, appears to be an effective strategy to treat pazopanib-induced liver toxicity, enabling patients who have shown to benefit from pazopanib, to continue this treatment safely.

### Supplementary Information

Below is the link to the electronic supplementary material.

Supplementary file1 (EPS 1169 KB)

Supplementary file2 (EPS 1175 KB)

Supplementary file3 (DOCX 15 KB)

Supplementary file4 (DOCX 19 KB)

## Data Availability

The data underlying this article will be shared on reasonable request to the corresponding author.
