# Supplementary material for: Real-world data on the management of pazopanib-induced liver toxicity in routine care of renal cell cancer and soft tissue sarcoma patients
Source: Cancer Chemother Pharmacol. 2023 Dec 17;93(4):353–64. doi: 10.1007/s00280-023-04615-7 (PMC10951019; doi:10.1007/s00280-023-04615-7)
Supplement: Supplementary file 3 — Supplementary file3 (DOCX 15 KB) [file 280_2023_4615_MOESM3_ESM.docx]

**Supplemental Material**

**Supplemental Method 1. Calculation of average pazopanib exposure.**

C_trough_ levels were calculated using the approach of Wang et al. [27]. In patients who used one dose level of pazopanib until the event, the average pazopanib concentration was calculated as the mean of the available pazopanib trough concentration (C_trough_) levels (sum of pazopanib C_trough_ levels / number of measurements).

In patients who used more than one dose level of pazopanib until the event, the average pazopanib concentration was calculated as follows: ((number of days of dose #1 • calculated C_trough_ level at dose #1) + (number of days of dose #2 • calculated C_trough_ level at dose #2)) / sum of days dose #1 and dose #2. An example: suppose a patient used 800mg pazopanib for 2 weeks and thereafter 600 mg for 6 weeks. At a dose of 800 mg the calculated C_trough_ was 35 mg/L and at the dose of 600 mg the calculated C_trough_ was 28 mg/L The average pazopanib concentration = ((14 • 35) + (42 • 28)) / (14 + 42) = 29.75 mg/L.

**Figure legends**

**Supplemental Figure 1. OS of patients with or without liver toxicity in patients with RCC (A) or STS (B).**

Kaplan-Meier curve of OS (in months) for patients who did (blue line) or did not (yellow line) experience liver toxicity during pazopanib treatment for metastatic RCC (median 38.5 versus 21.9 months; P=0.115) or STS (median 10.9 versus 12.6 months; P=0.593).

Abbreviations: OS, overall survival; RCC, renal cell carcinoma; STS, soft tissue sarcoma.

**Supplemental Figure 2. PFS of patients with or without liver toxicity in patients with RCC (A) or STS (B).**

Kaplan-Meier curve of PFS (in months) for patients who did (blue line) or did not (yellow line) experience liver toxicity during pazopanib treatment for metastatic RCC (median 13.8 versus 8.9 months; P=0.252) or STS (median 6.2 versus 5.8 months; P=0.376).

Abbreviations: PFS, progression free survival; RCC, renal cell carcinoma; STS, soft tissue sarcoma.
