# Supplementary material for: Real-world data on the management of pazopanib-induced liver toxicity in routine care of renal cell cancer and soft tissue sarcoma patients
Source: Cancer Chemother Pharmacol. 2023 Dec 17;93(4):353–64. doi: 10.1007/s00280-023-04615-7 (PMC10951019; doi:10.1007/s00280-023-04615-7)
Supplement: Supplementary file 4 — Supplementary file4 (DOCX 19 KB) [file 280_2023_4615_MOESM4_ESM.docx]

**Supplemental Table 1.** Management of individual patients with pazopanib-induced liver toxicity

| **Case number** | **Average pazopanib concentration before toxicity (mg/L)** | **Pazopanib concentration at toxicity (mg/L)** | **Pazopanib dose at toxicity** | **Treatment continuation after toxicity (if no, reason)** | **Treatment interrupted before continuation** | **Treatment with corticosteroids** | **Worst ALT level**  **(IU/l)** | **Recovery ALT? (if no, reason)** | **Pazopanib dose after toxicity** | **Duration pazopanib treatment after toxicity (days)** |
| --- | --- | --- | --- | --- | --- | --- | --- | --- | --- | --- |
| 1 | 26.0 | 26.0 | 800 mg fasted | No (switch treatment) | - | No | 375 | Yes | - | - |
| 2 | 35.7 | 35.7 | 800 mg fasted | No (PD) | - | No | 179 | No (referral to other hospital, lost to FU) | - | - |
| 3 | 10.9 | 11.1 | 600 mg + food | No (death) | - | Yes | 209 | No (death) | - | - |
| 4 | 29.6 | 29.6 | 600 mg fasted | No (switch treatment | - | Yes | 227 | Yes | - | - |
| 5 | 27.0 | 27.0 | 800 mg fasted | No (increased ALT + bilirubin) | - | Yes | 600 | Yes | - | - |
| 6 | 31.0 | 31.0 | 800 mg fasted | No (increased ALT + bilirubin) | - | Yes | 267 | Yes | - | - |
| 7 | - | - | 600 mg fasted | No (switch treatment) | - | Yes | 431 | Yes | - | - |
| 8 | 44.0 | 44.0 | 800 mg fasted | Yes | No | No | 154 | Yes | Same dose | 147 |
| 9 | 24.2 | 24.2 | 800 mg fasted | Yes | No | No | 133 | Yes | Equivalent dose (600 mg + food) | 369 |
| 10 | 27.7 | 64.2 | 800 mg + food | Yes | No | No | 116 | Yes | Dose reduction (600 mg + food) | 170 |
| 11 | 23.6 | 23.6 | 800 mg fasted | Yes | No | No | 111 | Yes | Same dose | 591 |
| 12 | 15.9 | 15.9 | 800 mg fasted | Yes | No | No | 150 | Yes | Dose increase (800 mg + food) | 50 |
| 13 | 45.7 | 45.7 | 800 mg fasted | Yes | No | No | 151 | Yes | Equivalent dose (600 mg + food) | 1282 |
| 14 | - | 35.5 | 800 mg fasted | Yes | No | No | 438 | Yes | Same dose | 505 |
| 15 | 31.5 | 31.5 | 800 mg fasted | Yes | Yes | No | 891 | Yes | Same dose | 567 |
| 16 | 41.2 | 41.2 | 400 mg fasted | Yes | Yes | No | 284 | Yes | Dose reduction (200 mg fasted) | 990 |
| 17 | 52.4 | 52.4 | 400 mg fasted | Yes | Yes | Yes | 385 | Yes | Dose reduction (200 mg + food) | 369 |
| 18 | 24.3 | 24.3 | 800 mg fasted | Yes | Yes | Yes | 225 | Yes | Same dose | 7 (recurrence liver toxicity) |
| 19 | - | - | 800 mg fasted | Yes | Yes | Yes | 452 | Yes | Dose reduction (600 mg fasted) | 200 |
| 20 | 22.0 | 22.0 | 800 mg fasted | Yes | Yes | Yes | 268 | Yes | Same dose | 66 |
| 21 | - | - | 800 mg fasted | Yes | Yes | Yes | 451 | Yes | Dose reduction (600 mg fasted) | 94 |
| 22 | 23.3 | 23.3 | 600 mg fasted | Yes | Yes | Yes | 141 | Yes | Same dose | 231 |
| 23 | 19.6 | 19.6 | 800 mg fasted | Yes | Yes | Yes | 878 | Yes | Dose reduction (600 mg fasted) | 114 |
| 24 | 62.0 | 62.0 | 800 mg fasted | Yes | Yes | Yes | 275 | Yes | Dose reduction (400 mg fasted) | 147 |
| 25 | 36.0 | 36.0 | 800 mg fasted | Yes | Yes | Yes | 436 | Yes | Dose reduction (600 mg fasted) | 6 (recurrence liver toxicity) |

Abbreviations: ALT, alanine transaminase; FU, follow-up; PD, progressive disease;
